# Application of Patient-Specific Computational Fluid Dynamics in Coronary and Intra-Cardiac Flow Simulations: Challenges and Opportunities

**DOI:** 10.3389/fphys.2018.00742

**Published:** 2018-06-26

**Authors:** Liang Zhong, Jun-Mei Zhang, Boyang Su, Ru San Tan, John C. Allen, Ghassan S. Kassab

**Affiliations:** ^1^National Heart Centre Singapore, National Heart Research Institute of Singapore, Singapore, Singapore; ^2^Duke-NUS Medical School, Singapore, Singapore; ^3^California Medical Innovations Institute, San Diego, CA, United States

**Keywords:** blood flow, computational fluid dynamics (CFD), patient-specific, cardiovascular, coronary, intra-cardiac flow simulation

## Abstract

The emergence of new cardiac diagnostics and therapeutics of the heart has given rise to the challenging field of virtual design and testing of technologies in a patient-specific environment. Given the recent advances in medical imaging, computational power and mathematical algorithms, patient-specific cardiac models can be produced from cardiac images faster, and more efficiently than ever before. The emergence of patient-specific computational fluid dynamics (CFD) has paved the way for the new field of computer-aided diagnostics. This article provides a review of CFD methods, challenges and opportunities in coronary and intra-cardiac flow simulations. It includes a review of market products and clinical trials. Key components of patient-specific CFD are covered briefly which include image segmentation, geometry reconstruction, mesh generation, fluid-structure interaction, and solver techniques.

## Introduction—coronary artery disease, cardiac dysfunction, and diagnosis

In coronary artery disease (CAD) atherosclerotic build-up can narrow the arterial lumen, resulting in myocardial ischemia. Prevalence of CAD is 6% in the general population and up to 20% in those aged over 65 years. About 13% of deaths are due to CAD. By 2030, it is projected that 15% of male deaths will be attributable to CAD (World Health Organization, 2011).

CAD can be diagnosed by means of either an anatomic parameter, such as diameter stenosis or a functional parameter linked to coronary territory myocardial ischemia. Stenosis does not invariably impair distal coronary flow, and this is particularly true with regard to the intermediate coronary artery lesions (i.e., diameter stenosis between 30 and 70%). Non-invasive tests of myocardial ischemia (e.g., nuclear myocardial perfusion imaging, stress echocardiography) identify areas of the most severely reduced relative coronary flow reserve. They are fairly accurate for myocardial ischemia detection on a per-patient basis, but these perform less well in quantifying severity of individual coronary territory ischemia. The latter is relevant in multi-vessel percutaneous coronary intervention (PCI), where coronary physiological information, overlaid on detailed maps of patient-specific coronary artery anatomy, dictates management decisions. Fractional flow reserve (FFR) measured during invasive coronary angiography (ICA) under adenosine-induced hyperemia has emerged as the gold standard for assessment of coronary flow physiology and coronary territory ischemia (Johnson et al., 2012).

Diagnosis of heart contractile dysfunction requires demonstration of either diastolic or systolic function abnormalities. The gold standard for determining diastolic dysfunction is an increase in invasively measured ventricular end-diastolic pressure–>15 mmHg in the case of the left ventricle (LV) (Nishimura and Tajik, 1997). Systolic dysfunction is assessed by the change in maximal ventricular pressure (P) during isovolumic contraction, dP/dt_max_ (Yamada et al., 1998). Multiple ventricular pressure-volume loops assayed using a conductance catheter under varying loading conditions can yield end-diastolic (E_ed_) and end-systolic elastances (E_es_) that characterize ventricular diastolic and systolic dysfunction, respectively (Burkhoff et al., 2005). Emerging noninvasive echocardiographic and cardiac magnetic resonance (MRI) imaging techniques enable corroborative assessment of regional and global cardiac chamber dysfunction involving strain and strain rate (Zhong et al., 2012), curvedness (a descriptor of three-dimensional ventricular shape) and curvedness rate (Zhong et al., 2009), ventricular contractility dσ^*^/dt_max_, where σ^*^ is pressure-normalized wall stress (Zhong et al., 2014), and atrio-ventricular velocities (Leng et al., 2016).

Patient-specific computational fluid dynamics (CFD) modeling is a recent development. Non-invasive FFR (FFR_CT_) is derived from CFD modeling of images acquired using computed tomography coronary angiography (CTCA). With invasive FFR as the gold standard, FFR_CT_ ≤ 0.80 is superior to both CTCA and ICA determined diameter stenosis for ascertaining ischemia on a coronary artery territory basis (Min et al., 2012). FFR_CT_ analysis is solely available via a centralized commercial web-based service of the HeartFlow® company. Time-consuming computational demands and high costs−6 h and $2000 USD to process a case (Kimura et al., 2015)—hamper widespread clinical adoption. The requisite offsite handling of sensitive confidential patient information and associated medical conditions is a highly delicate issue involving IT-security, potential for data abuse, etc.

Unlike coronary blood flow simulation, CFD studies on intra-cardiac flows are primarily confined to research purposes owing to the complexity of modeling intra-cardiac flows. In truth, coronary and intra-cardiac flows are closely connected. Coronary artery dysfunction leads to myocardium ischemia, and intra-cardiac flows provide blood for circulation throughout the body, including the coronary circulation. Prolonged and untreated myocardial ischemia could increase the risk for death or myocardial infarction (Iskander and Iskandrian, 1998). Future integration of both coronary and intra-cardiac flow simulations is desirable to enable a comprehensive assessment of cardiac circulatory pathophysiology. This paper aims to pave the way for integrated simulations and focuses on a progress review of CFD applications in modeling coronary and intra-cardiac flow. Other applications of CFD in cardiovascular disease can be found in Morris et al. (2015).

## Coronary flow simulation

### Challenges and opportunities in patient-specific simulation techniques for studying blood flow in coronary arteries

**Figure 1:**
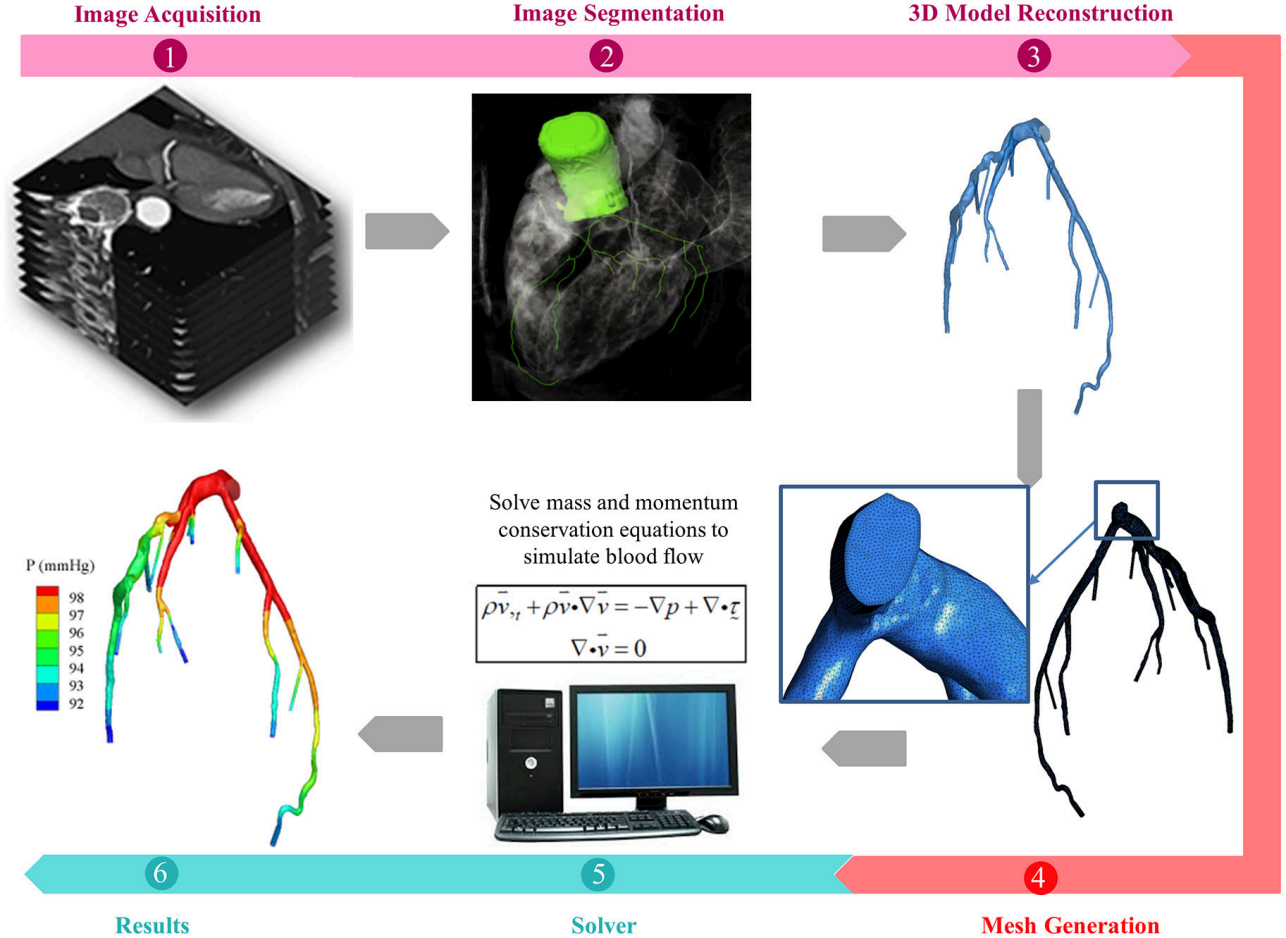
Schematic drawings for the procedures of patient-specific simulations for blood flow in coronary arteries, including (1) acquisition of CTA or ICA images (2) segmentation of acquired images (3) reconstruction of 3D model (4) mesh generation to discretize the 3D model (5) solving the mass and momentum conservation equations to simulate the blood flow in coronary arteries, if FSI is taken into consideration, solid solver will also be activated, and (6) presentation of simulations.

In general, the tasks involved in performing CFD simulation for a patient-specific coronary artery tree are as follows: (1) Image acquisition and segmentation to reconstruct a 3D patient-specific coronary model; (2) CFD preprocessing to discretize the domain with meshes and define the boundary conditions; and (3) Solving the fluid governing equations using a fluid solver and post-processing to visualize the flow field (Figure 1). If fluid structure interaction (FSI) is considered, an additional solid solver is used, and coupling between fluid and solid solvers is implemented. Table 1 summarizes the challenges involved.

**Table 1:** Current challenges and opportunities in numerical simulation of coronary arteries.

**Simulation procedures**		**Current challenges and opportunities**
Image acquisition		•Current spatial resolution for CTCA and ICA was around 0.3 mm (Kantor et al., 2007; Lewis et al., 2016; Galassi et al., 2018). This limits their use to coronary arteries of 1 mm or greater in diameter. The ideal spatial resolution is 0.1 mm (Lewis et al., 2016). Severe motion artifacts, stair-step artifacts, image noise, or calcium blooming may lead to non-diagnostic CTCA images (Alkadhi et al., 2008). Image resolution and quality can be mitigated to an extent by extracting images in different cardiac phases and using multiple imaging modalities for reconstruction (Sankaran et al., 2016).
Segmentation and 3D model reconstruction		•It is challenging to segment images with severe motion and stair-step artifacts, image noise, calcification, or misregistration. •Segmentation of coronary artery tree may take a few hours.
Fluid dynamics simulation	Fluid mesh generation	•Quality control of tetrahedral meshes can be challenging (Wittek et al., 2016). Meshless methods discretize the computational domain into a cloud of nodal points (Belinha, 2016), which may allow direct model generation from CTCA or MRI images.
	Boundary conditions	•Both prescribed and lumped parameter (0 or 1-order) models can be used as boundary conditions. The lumped parameters (e.g., resistance, compliance, etc.) may be tuned via numerical optimization (Spilker et al., 2007).
	Fluid solver	•Both robust implicit approaches and explicit methods can be used to solve the flow-governing equations.
		•Explicit methods are generally less robust compared to the implicit fully coupled methods (Kim et al., 2010; Sankaran et al., 2012).
FSI coupling		•Traditional FSI techniques based on ALE method requires expensive computational cost due to re-meshing (Hecht and Pironneau, 2017). Immersed boundary (Peskin, 2003) and coupled momentum methods (Figueroa et al., 2006) are alternatives to treat coronary vessels as compliant.

Coronary anatomy can be imaged using ICA, intravascular ultrasound (IVUS), optical coherence tomography (OCT), CTCA, and MRI (Zhang et al., 2014). Invasive IVUS and OCT yields high-resolution cross-sectional views of the coronary arteries, and can be used in conjunction with biplane ICA to reconstruct the 3D vessel model. Since non-invasive CTCA possesses higher spatial resolution than MRI and echocardiography, it is widely used for 3D patient-specific coronary model reconstruction. However present CTCA has a spatial resolution of about 0.3 mm, which limits its use to coronary arteries of 1 mm or greater in diameter. Although CAD is not generally characterized nor is FFR measured in such small vessels, the latter is essential for characterizing coronary microcirculation.

Sophisticated segmentation approaches such as level-set segmentation (Bekkers and Taylor, 2008) have been applied to reconstruct 2D and 3D patient-specific coronary models, either by fusion of biplane ICA with IVUS images (Papafaklis et al., 2007) or directly from CTCA images (Torii et al., 2009). Commercial (e.g., 3D Doctor, Mimics, SliceOmatic, Amira) and open-source general image processing tools [e.g., VTK, ITK, ITK-SNAP, VTK, Analyze, and ImageJ (or Fiji)], make reconstruction of patient-specific models from medical images possible. Furthermore, their plug-in capability allows easy customization of segmentation tools. Artifacts such as calcification, motion and mis-registration are not easily overcome by segmentation techniques, and remain challenging.

In terms of simulation tools, commercial software such as ANSYS (including ICEM, FLUENT, CFX), STAR-CCM, and open-source tools (e.g., OpenFOAM) is applicable to general CFD simulations, including simulating the blood flow in coronary arteries. SimVascular (Schmidt et al., 2008) is a special tool designed for simulating the blood flow in vessels. These tools allow users specifications on mesh generation, boundary conditions settings and etc. As regards meshes, mesh generation schemes can be classified as structured or unstructured meshes. Structured grid generators, including “block-structured” techniques (used in ICEM CFD, TrueGrid, and IA-FEMesh) generally require complex iterative smoothing procedures to align elements with boundaries or physical domain. For the complex 3D coronary artery models reconstructed from medical images, unstructured meshes are commonly needed, which are built based on node coordinates and the connections between nodes to form elements. Commercial packages (e.g., ANSYS, TGrid) and open-source (e.g., TetGen, gmesh) allow automatic discretization of complex geometry with tetrahedral meshes. However quality control of tetrahedral meshes can be challenging and varies according to the mesh generation method employed (Wittek et al., 2016). The advancing front method, such as the Delaunay triangulation method, can provide better control of the mesh quality, but at the expense of prolonged computational time. In addition, 4-noded tetrahedral elements are involved with artificial stiffening, which presents challenges in modeling soft tissue, such as the coronary artery wall (Wittek et al., 2010). Higher-order and mixed-formulation tetrahedral elements can assist in overcoming these challenges. Nevertheless their computational cost is about four times higher than the 4-noded tetrahedral elements (Bourdin et al., 2007). To overcome the difficulty of generating good quality meshes for complex geometry with limited time and the convergence difficulties in modeling structures with large deformations, meshless methods have been recognized as one possible solution (Doblare et al., 2005). Meshless methods discretize the computational domain into a cloud of nodal points (Belinha, 2016). This discretization flexibility may allow direct model generation from CTCA or MRI images. However, meshless methods also have substantial shortcomings: (i) limited strict mathematical proof, (ii) incomplete theory, and (iii) lower computation efficiency compared to traditional computational methods using meshes (Zhang et al., 2012).

To achieve an acceptable simulation of blood flow in the coronary arteries, proper boundary conditions are paramount. Although flow and pressure waveforms can be obtained from the literature, *in-vitro* and *in-vivo* measurements are necessary for accurate simulations. For many CFD applications, it is virtually impossible to know flow and/or pressure waveforms a *priori* due to the difficulty of obtaining simultaneous measurements in coronary arteries. To solve this problem, multi-scale simulations have been developed that couple 3D simulation with reduced-order (1 or 0-dimensional) models at the boundaries. These models characterize pressure and flow rate in upstream and downstream vasculatures (Kim et al., 2010) as resistance, compliance, and impedance. How to determine the values of these patient-specific parameters remains a dilemma. Although morphologic information (e.g., scaling law) is widely used, numerical optimization may be necessary to tune these patient-specific parameters (Spilker et al., 2007).

Another obstacle in multi-scale simulation is how to solve the flow-governing equations using reduced-order models as boundary conditions. Both robust implicit approaches and explicit methods have been used. Explicit methods do not require changing the numerical algorithms to solve the governing equations (Sankaran et al., 2012), although they are generally less robust compared to the implicit fully coupled methods (Kim et al., 2010).

Recent progress in FSI techniques has allowed treating the coronary vessels as compliant. In the traditional Arbitrary Lagrangian-Eulerian (ALE) method (Malvè et al., 2012), boundaries and interfaces of both fluid and structural computational domains are precisely tracked during the iterations. When taking heart movement into consideration, re-meshing computational domains is often necessary to maintain mesh quality, which substantially increases computational cost. Over the years, stability of the ALE method has been improved (Hecht and Pironneau, 2017).

Alternative FSI techniques used to simulate flow in the presence of a moving boundary include immersed boundary and coupled momentum methods. These use fixed fluid meshes with boundaries defined by a set of moving Lagrangian points (Peskin, 2003) or linear membrane (Figueroa et al., 2006). Although prescribed heart motion has been used for simulating the blood flow in left (Prosi et al., 2004) and right (Torii et al., 2010) coronary arteries, more efficient, and robust FSI techniques are necessary to model large deformations of coronary arteries when taking heart motion during a cardiac cycle into account.

Recent development of fluid-solid-growth modeling incorporates vascular wall geometry, structure and properties governed by stress-mediated growth and remodeling (G&R) into FSI simulation (Figueroa et al., 2009). In this way, biofluid mechanics, biosolid mechanics, and biotransport phenomena, such as arterial growth, remodeling, and adaptation (Valentín et al., 2013) can be better understood. Recently, machine learning has been adopted into the CFD simulation to reduce the computational time incorporating features trained by a database generated from a set of offline CFD simulations (Sankaran et al., 2015; Itu et al., 2016). Sankaran and Marsden (2011) developed an adaptive collocation algorithm to quantify the effect of input uncertainties on cardiovascular simulation. Using a data-driven framework, Sankaran et al. (2016) studied the impact of anatomic and physiologic uncertainty (e.g., various boundary conditions and blood viscosity) on blood flow simulation. These data-driven modeling approaches wherein CFD data is used to enrich and refine the models may become popular in the future.

In addition, the assumption of blood as a Newtonian fluid is only valid for shear rate higher than 100 s^−1^, which may not be true when a flow recirculation region is formed near a coronary stenosis. The influence of non-Newtonian properties of blood on the velocity distribution and shear-thinning has been studied via various single and multi-phase non-Newtonian hemodynamic models (Jung et al., 2006). An effect of non-Newtonian properties on overall pressure drop across the arterial stenosis was exhibited at a flow with the Reynolds number of 100 or less (Cho and Kensey, 1991).

### Challenges and opportunities in applying patient-specific CFD technologies for diagnosing the severity of coronary artery disease

**Figure 2:**
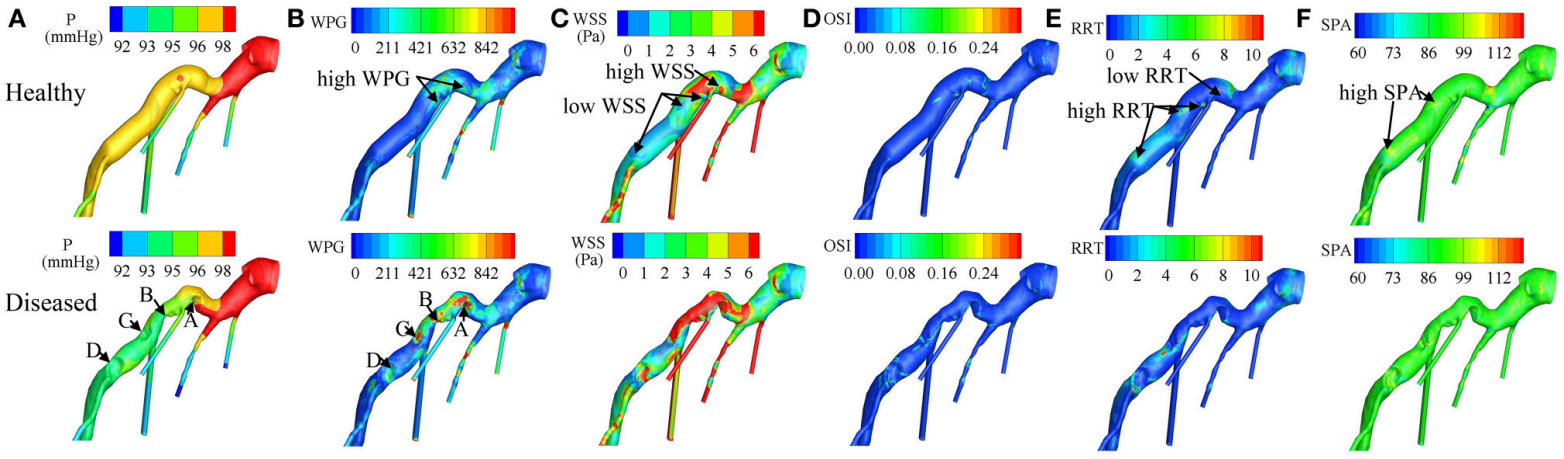
Distributions of **(A)** P (Pressure), **(B)** WPG (wall pressure gradient), **(C)** WSS (wall shear stress), **(D)** OSI (oscillatory shear index), **(E)** RRT (relative residence time), and **(F)** SPA (stress phase angle) on the virtually healthy and diseased left coronary artery trees respectively. Labels of **(A–D)** indicate the stenosis locations. In the virtually healthy artery model, low WSS, and high RRT was exhibited in three of the four locations, where the stenoses were formed, and high WSS with low RRT was exhibited in the fourth. These findings suggest that coronary plaque is more likely to form in locations with low- WSS- and- high- RRT or high- WSS- and- low- RRT. From Zhang et al. (2015).

Recent patient-specific CFD simulations provide detailed hemodynamic parameter (HP) information, such as pressure (P), wall pressure gradient (WPG) (Liu et al., 2012), wall shear stress (WSS) (Papafaklis et al., 2007; Stone et al., 2012), oscillatory shear index (OSI), relative residence time (RRT), and stress phase angle (SPA) (Knight et al., 2010), enabling characterization of HP distributions on coronary vessel walls. Definitions of these HPs (Table 2) and illustrations of their distributions in a left coronary artery tree (Figure 2) are presented. In the simulations, pressure and resistance boundary conditions were assigned to the inlet and outlets, respectively. A non-slip condition was imposed on the wall (Zhang et al., 2015).

**Table 2:** Hemodynamic parameters (HPs) predicted by CFD (Computational Fluid Dynamics) to link with CAD (Coronary Artery Disease).

**HPs**	**Units**	**Definition and Formula**	**Related hypothesis**	**Related studies**	**Remark**
P	Pa	Force acting perpendicularly on the vessel wall per unit area	Elevated blood pressure is associated with atherosclerotic formation (Glagov et al., 1989)	Aueron and Gruentzig, 1984	FFR is the pressure based indicator for CAD diagnosis (Johnson et al., 2012). Recent coupling between CFD and medical images leads to new indictors, such as FFR_CT_, FFR_QCA_, _C_FFR and FFR_B_ (Nørgaard et al., 2014; Tu et al., 2014; Coenen et al., 2015; Zhang et al., 2016, 2018)
WPG	Pa/m	Spatial gradient of the wall pressure $WPG=\sqrt{{\left(\frac{\partial P}{\partial x}\right)}^{2}+{\left(\frac{\partial P}{\partial y}\right)}^{2}{+\left(\frac{\partial P}{\partial z}\right)}^{2}}$ *Note: P* is pressure exerted on the wall; *x, y* and *z* are coordinates in different directions	WPG may represent important local modulators of endothelial gene expression in atherogenesis and may result in the redistribution of the initially accumulated atheromatic material within the sub-endothelial layer	Liu et al., 2012	
WSS	Pa	Frictional force of blood exerted tangential to the luminal surface of the blood vessel per unit area $wss=\tau_{w}=\;\mu \left(\frac{\partial \vec{u}}{\partial \vec{n}}\right){|}_{wall}$ Note: $\vec{u}$ and $\vec{n}$ are the velocity vector and the direction vector normal to the wall, respectively	WSS over normal coronary artery was found to within the range of 15–20 dynes/cm^2^ a. High WSS is conjectured to injure and denude the vessel wall of endothelial cells, resulting in atherosclerotic plaque (Fry, 1968) b. Low WSS is suspected to prolong particle retention time and increase intimal accumulation of lipids, leading to atherosclerosis formation (Caro et al., 1969)	Combing CFD with IVUS images and biplane coronary angiography helps to predict WSS, which is correlated with baseline luminal narrowing or plaque thickness (Stone et al., 2003; Papafaklis et al., 2007; Gijsen et al., 2008); Habib (Eshtehardi et al., 2012; Stone et al., 2012)	Among them, PREDICTION study (Stone et al., 2012) is impressive. Study recruited 506 patients. Results revealed that low local WSS and large plaque burden could identify plaques that develop progressively and lead to lumen narrowing
OSI		A measure to quantify the change in direction and magnitude of the WSS (Ku et al., 1985) $OSI=\frac{1}{2}\left(1-|\int_{0}^{T}\tau_{w}dt|/\int_{0}^{T}|\tau_{w}|dt\right)$ *Note: T* is the duration of one cardiac cycle	Marked oscillations in the direction of WSS could be captured by high OSI values, which may lead to atherogenesis (Knight et al., 2010)	Knight et al. (2010) obtained CT images of 30 patients with 120 plaques. By virtually removing the plaques, CFD predicted OSI was correlated with plaque location	It was found that OSI has higher positive prediction value (PPV) than WSS
RRT	Pa^−1^	The residence time of a particle in the vicinity of vascular endothelium (Himburg et al., 2004) $RRT=\frac{1}{\left(1-2\times OSI\right)\times TAWSS}$ Note: TAWSS is the time-averaged WSS	Prolonged residence time of blood, viz. higher RRT, may increase the likelihood of adhesion of platelets and leukocytes to the endothelium and lead to the smooth muscle cell proliferation	Kleinstreuer et al., 2001; Knight et al., 2010	It was found that RRT had higher PPV than WSS
SPA		Time-averaged temporal phase angle between circumferential stress (CS) and WSS on the arterial wall to quantify the time lag arises between the pulsatile WSS and CS (Qiu and Tarbell, 1996) *SPA* = φ(*D*−τ_*w*_) Note: φ(*D*−τ_*w*_)is the time-averaged phase difference between lumen diameter circumferential direction and WSS	SPA measures the degree of asynchrony between pressure and flow waveforms	Torii et al., 2009; Zhang et al., 2015	SPA is proposed to be a useful indicator in predicting sites prone to atherosclerosis

Among the HPs, pressure was closely related to FFR (Johnson et al., 2012), where FFR is the ratio of pressure distal to stenosis and aortic pressure at hyperemia, obtained during ICA. Alternative approaches for calculating FFR non-invasively have been attempted by several groups.

**Figure 3:**
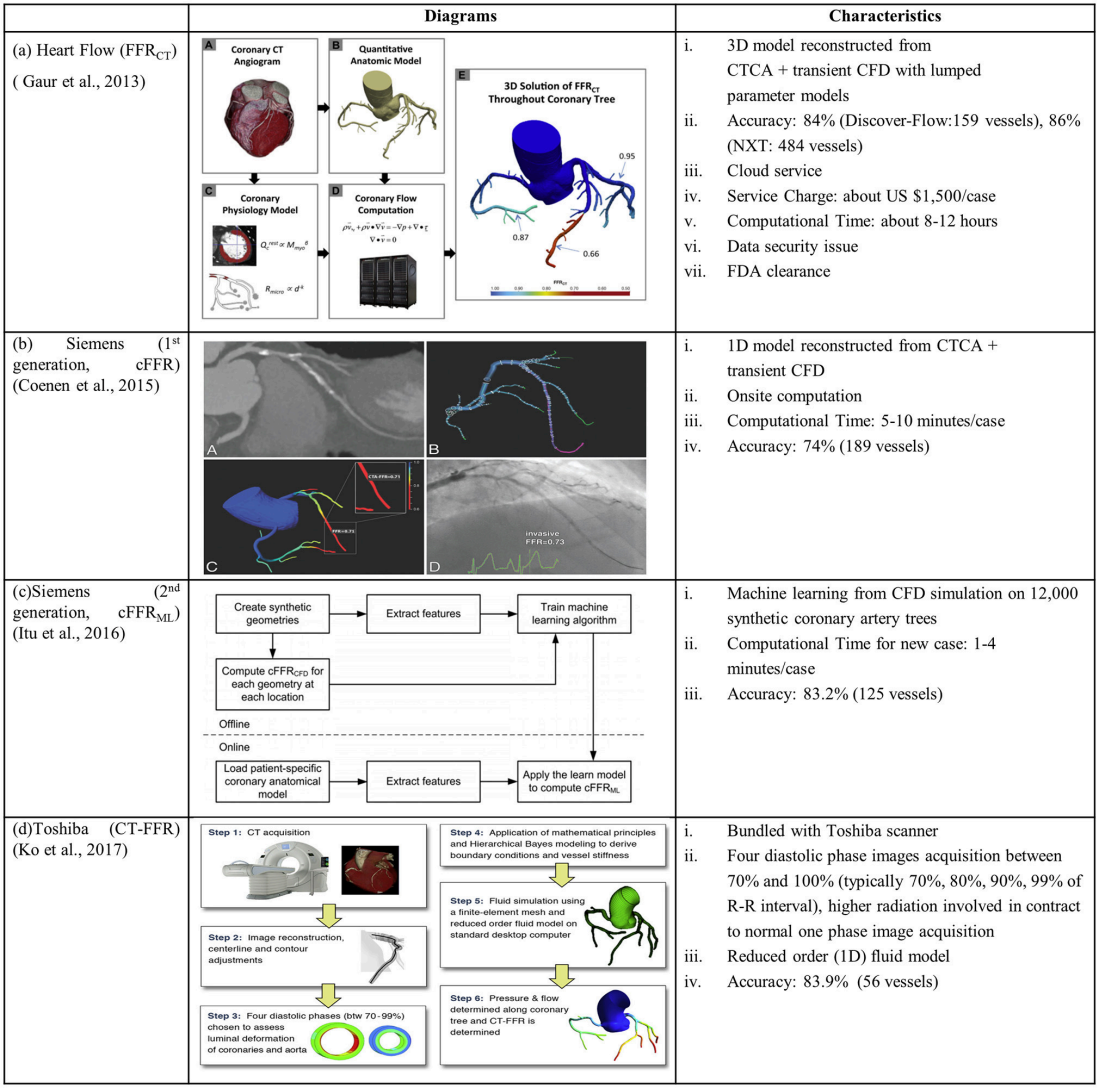
Diagrams and characteristics for calculating non-invasive FFR through combining CTCA with CFD by the companies of **(A)** Heart Flow: FFR_CT_ (Gaur et al., 2013); **(B)** Siemens (1st generation: cFFR) (Coenen et al., 2015); **(C)** Siemens (2nd generation: cFFR_ML_) (Itu et al., 2016); and **(D)** Toshiba: CT-FFR (Ko et al., 2017).

The Heart Flow company is a pioneer to combine CTCA images with CFD for calculating non-invasive FFR_CT_ in CAD diagnosis. Kim et al (Kim et al., 2010) pioneered non-invasive FFR_CT_ by reconstructing 3D patient-specific coronary artery model from CTCA images and coupling lumped parameter models to an implicit solver of fluid-governing equations (Figure 3A). Multicenter clinical trials DISCOVER-FLOW, DeFACTO, and NXT (Koo et al., 2011; Min et al., 2012; Nørgaard et al., 2014) demonstrated superior diagnostic accuracy for FFR_CT_ vs. CTCA alone. PLATFORM trial (Douglas et al., 2015) (Lu et al., 2017) demonstrated the feasibility and safety of FFR_CT_ as a diagnostic strategy in triage of patients with suspected CAD compared to standard of care. REAL-FFR_CT_ (Kawaji et al., 2017) demonstrated good diagnostic performance of FFR_CT_ even in patients with severely calcified vessels. Recently HeartFlow's FFR_CT_ software has been approved by FDA for measuring coronary blockages non-invasively. However it used cloud technology for uploading CTCA images and downloading FFR_CT_ report. This could involve data security issues that would hinder the on-site computation.

To facilitate on-site FFR computation supported by Siemens Company, Coenen et al. modeled coronary vessel as 1D segment and employed a reduced-order model for simulating the coronary circulation. In this way, the computational time was reduced to 5–10 min per patient (Coenen et al., 2015). Calculated pressure information, viz. computational FFR (cFFR), was mapped onto a 3D model reconstructed from CTCA images (Figure 3B). The correlation between cFFR and FFR, however, was poor (*r* = 0.59). To further reduce the computational time, Itu and colleagues from Siemens applied machine-learning approach for computing cFFR_ML_ with features extracted from training database (Itu et al., 2016). The database consisted of synthetically generated coronary artery models and corresponding FFR values computed from the CFD algorithm (Figure 3C).

Another attempt to reduce the computation time to be < 30 min was conducted by Ko and colleagues from Toshiba Medical Systems Corp. core laboratory (Ko et al., 2017). Differing from the above studies, 4 CTCA images were acquired and reconstructed at phases of 70, 80, 90, and 99% of the R-R interval. The arterial luminal deformation was taken into consideration and a reduced-order fluid model was used to simulate a 1-dimensional pressure and flow distribution in coronary tree (Figure 3D). In this approach, the interaction between fluid and structure is taken into account to some extent, although it is not a fully-coupled FSI simulation.

With the exception of CTCA images, attempts have been made to derive FFR_QCA_ using CFD simulation in patient-specific coronary artery models reconstructed from ICA In a study involving 77 coronary vessels (Tu et al., 2014), FFR_QCA_ correlated well with the gold standard FFR (*r* = 0.81, *p* < 0.001). Invasive FFR_QCA_ obviates the need for pressure wire/catheter and adenosine. QFR was further derived from 3 flow models using fixed empiric flow velocity; modeled hyperemic flow velocity derived from measured angiography without administration of adenosine, and measured hyperemic flow velocity, respectively (Tu et al., 2016). Diagnostic accuracy of QFR was tested in the FAVOR II China (Xu et al., 2017) and WIFI II (Westra et al., 2018) studies. Based on this method, Medis QAngio 3D XA software was developed to calculate QFR.

Combining coronary angiogram images with CFD simulation was also studied by Morris et al. (2013) to estimate virtual fractional flow reserve (vFFR) with generic boundary conditions. VIRTUheart software was therefore developed to facilitate the calculation of vFFR. CathWorks is another tool available for FFR simulation-based service through the combination of coronary angiograms and CFD simulation.

Infusion OCT with a coronary angiogram was used by Poon et al. (2015, 2017) in an attempt to reconstruct the vessel and calculate virtually derived FFR.

Other HPs (Table 2) have been considered in relation to CAD based on biomechanical forces involved in regulation of blood vessel structure (Langille and O'Donnell, 1986). Among them, WSS has been the most studied. WSS is the frictional force of blood exerted tangentially to the luminal surface of the blood vessel per unit area. WSS is typically within the range of 15–20 dynes/cm^2^ (Kassab and Navia, 2006) for normal arteries; abnormal WSS outside this range promotes atherogenesis. There are two competing theories. In *in-vitro* experiments on canine thoracic aorta endothelial cells (ECs), ECs became abnormal for WSS >379 dynes/cm^2^ (Fry, 1968). This implied that high WSS might injure and denudate the ECs resulting in atherogenesis. Conversely, Caro et al. found intimal thickening and atherosclerosis with WSS < 6 dynes/cm^2^ (Caro et al., 1969). They conjectured that low WSS was associated with prolonged particle retention time, and increased intimal accumulation of lipids, leading to atherogenesis. Indeed, Rutsch et al. proposed several signaling pathways relating disturbed WSS with EC mechanico-chemical transductions (Rutsch et al., 2011).

CFD has been applied in the study of HP distributions, particularly WSS, in both idealized and patient-specific coronary artery models (Papafaklis et al., 2007). The PREDICTION study (Stone et al., 2012) enrolled 506 PCI patients and studied the natural history of plaque development in a subset of 374 (74%) over a 6- to 10-month period post-PCI. WSS was calculated for 3D coronary artery models reconstructed by combining intracoronary IVUS and biplane ICA images. Large plaque burden and low local WSS were found to be independent and additive predictors of plaque progression and luminal narrowing. Other clinical trials are currently underway investigating potential links between HPs and coronary atherogenesis (Antoniadis et al., 2015). These presage exciting possibilities for identifying indicators that may be useful for CAD diagnosis and patient management.

## Intra-cardiac flow simulation

### Challenges and opportunities in patient-specific simulations for studying intra-cardiac flow

**Figure 4:**
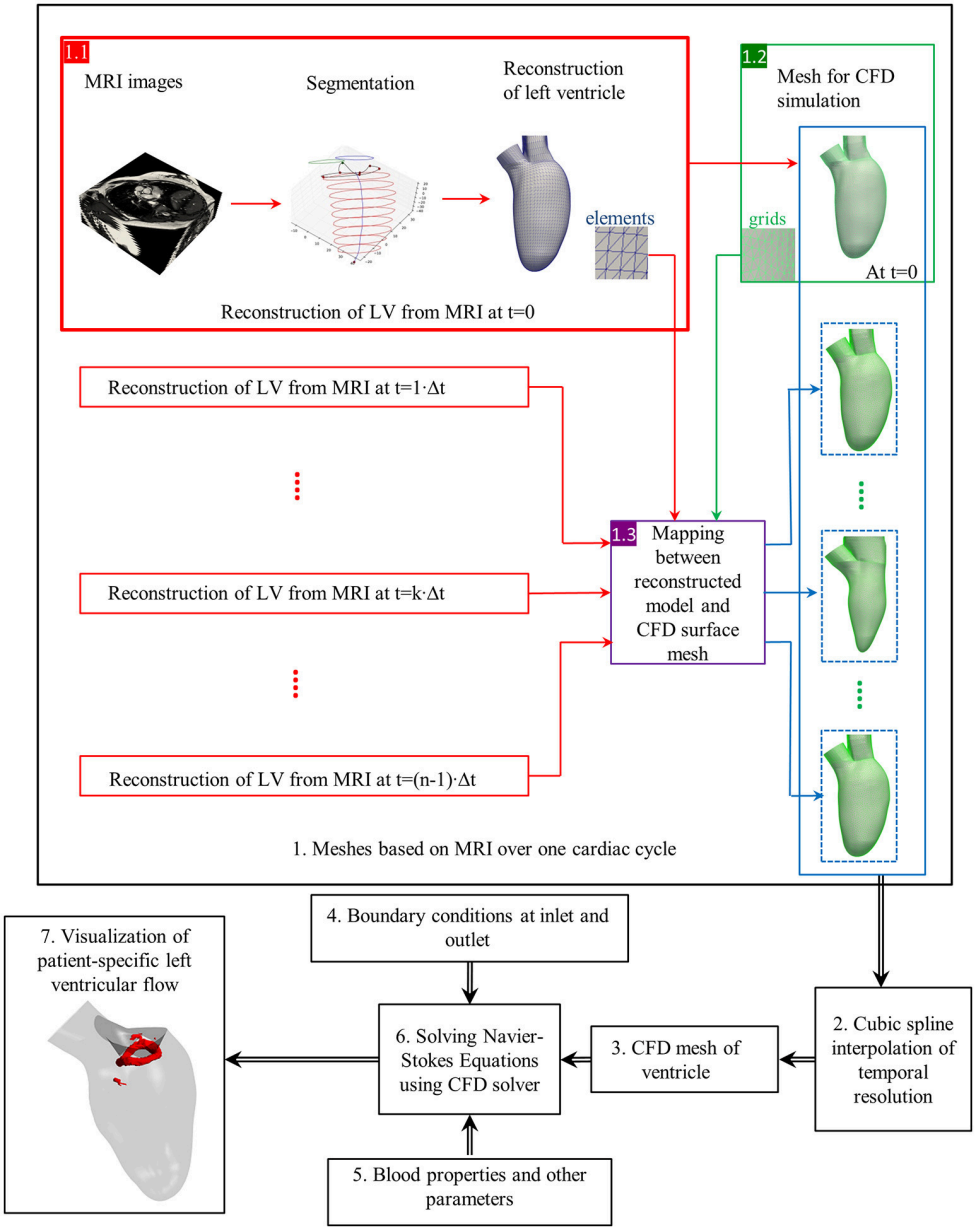
Flow chart of CFD simulation of patient-specific intra-cardiac flow. Black box: each step of numerical simulation (Su et al., 2016). Red box: left ventricle reconstruction from MRI images. Green box: CFD mesh generation. Violet box: Mapping between reconstructed model and CFD surface mesh. Dotted blue box: CFD mesh resulted from mapping. Blue box: A series of CFD meshes at each frame. Elements (blue) and grids (green) are for reconstructed geometry and CFD mesh, respectively.

Figure 4 illustrates the general process of CFD simulation of blood flow in the LV based on cardiac MRI images (Su et al., 2015). Images comprise the long-axis and a stack of 12–14 short-axis images covering the LV from apex to base. Typical slice thickness is 8 mm; and typical frame rate 20–40 per cardiac cycle. Short-axis images are segmented either manually or automatically wherein blood pool is distinguished from heart muscle, and papillary muscles are included in blood pool. Long-axis images are used to track the mitral annulus at the intersection of the LV and the left atrium, which cannot be segmented easily on short-axis images due to through-plane displacement. A similar method is applied to construct the aortic annulus at the LV outflow tract. A patient-specific model based on segmentations is thereby generated (steps are highlighted by red rectangle in 1.1 of Figure 4).

Next, the complex 3D models are discretized into tetrahedral, hexahedral or polyhedral grids using either in-house or commercial mesh generators such as ANSYS ICEM CFD, STAR-CCM+. A tetrahedral mesh is frequently adopted, which requires re-meshing when spring-based smoothing fails to cope with large deformations. Polyhedral meshes confer superior convergence speed (Spiegel et al., 2011) and can be easily implemented for FSI simulation. To factor in wall motion during numerical simulation, surface mesh numbers and their connectivity must match at each time step. By exploiting consistent topology within the patient-specific model, surface meshes at other time frames are generated based on the corresponding LV geometry, and the correlation between the 3D model and the surface mesh at the first time step. Cubic-spline interpolation is commonly applied, as the frame rate of MRI is inadequate for numerical simulation.

In simulations that use the immersed boundary method (Peskin, 2003), a Cartesian mesh is typically applied to the blood domain, where the LV surface consists of triangular facets. In other words, the discretization step can be skipped by using the patient-specific model directly.

Boundary conditions at inlet and outlet are initialized with appropriate blood properties prior to solving the Navier-Stokes equations. Cardiac flow profiles measured from velocity-encoded imaging modalities such as phase-contrast MRI scans can be used to specify inlet and outlet boundary conditions (Wong et al., 2017). Aortic/pulmonary pressure can only be measured invasively, which might not always be possible.

In addition, the heart is a complex multi-scale system involving the interaction of cardiac electrophysiology with muscle tissue, rapid valve opening and closure, and large wall deformation during the cardiac cycle (Quarteroni et al., 2017). To consider the coupling between electrophysiology and heart mechanics, fiber orientation/ architecture information is necessary for modeling electrical conduction and associated force generation (Crozier et al., 2016). As *in-vivo* acquisition of the fiber architecture is difficult (Toussaint et al., 2013), “Rule-based” methods was used to assign fiber orientation to ventricular cardiac models, which did not include the fiber structure within endocardial and intramural structures (Bayer et al., 2012). “Atlas-based” approaches used a mesh warping process to map the meshes associated with diffusion tensor MRI fiber data on an idealized template mesh. In this way, the fiber architecture information can be automatically incorporated into new patient-specific model (Vadakkumpadan et al., 2012).

The appropriate choice of constitutive models and material parameters to represent valves in computational studies is another topic related to the heart valve mechanics. In the study of Rausch et al. (2013), mitral valves were modeled as follows: (i) the Neo-Hooken isotropic nonlinear hyperplastic model (neglecting the anisotropic microstructure of mitral leaflet tissue; (ii) the coupled May-Newman model to characterize the heterogeneous response of the entire mitral valve complex; (iii) the decoupled Holzapfel model to represent the anisotropic properties of arterial tissue. The last two models resulted in smaller local displacement errors relative to the first model.

Cardiac tissue properties are the other important parameters for modeling the cardiac multi-scale interaction. However *in-vitro* measurement of tissue properties using a cardiac tissue mechanics testing system (Golob et al., 2014) is not applicable to the circumference in most cases. Estimating the material stiffness from pressure-volume loop analysis might be a practical way (Pironet et al., 2013). Modeling flow-mediated thrombus generation becomes feasible by coupling the hemodynamic equations to the biochemical convection–diffusion–reaction (CDR) equations (Mittal et al., 2016). Challenges of patient-specific intra-cardiac flow simulation are summarized in Table 3.

**Table 3:** Current challenges and opportunities in numerical simulation of intra-cardiac flow.

**Simulation procedures**		**Current challenges and opportunities**
Image acquisition		•Current spatial and time resolution for cardiac MRI was around 1–1.5 mm/40–50 ms respectively (Saeed et al., 2015), which are inadequate for assessing the rapid opening and closing of thin heart valves.
Segmentation and 3D model reconstruction		•Segmentation and 3D model reconstruction of the valves and right ventricle is challenging due to the limited spatial resolution of current MRI technology.
Fluid dynamics simulation	Fluid mesh generation	•To factor in wall motion during numerical simulation with dynamic meshes, the number of surface meshes and their connectivity must match at various time frames. Cubic-spline interpolation is usually needed to achieve adequate number of meshes for transient numerical simulation. This might be challenging for a complex heart chamber with valves, especially for the patients with heart disease.
		•Cartesian meshes can be used when the blood flow is simulated using the immersed boundary method (Peskin, 2003).
	Boundary conditions	•Realistic pressure and flow information could be provided through phase-contrast MRI, cardiac catheterization and etc.
	Fluid solver	•Improvement of computational speed to solve complex flow phenomena for heart chamber and valves are essential for the multi-physics coupling.
Multi-physics coupling and others		•Besides FSI, coupling electrophysiology with mechanics is also important in understanding heart function (Quarteroni et al., 2017) The definitions of fiber orientation (Crozier et al., 2016) and tissue properties (Golob et al., 2014) are important for modeling the cardiac multiscale interaction.

**Figure 5:**
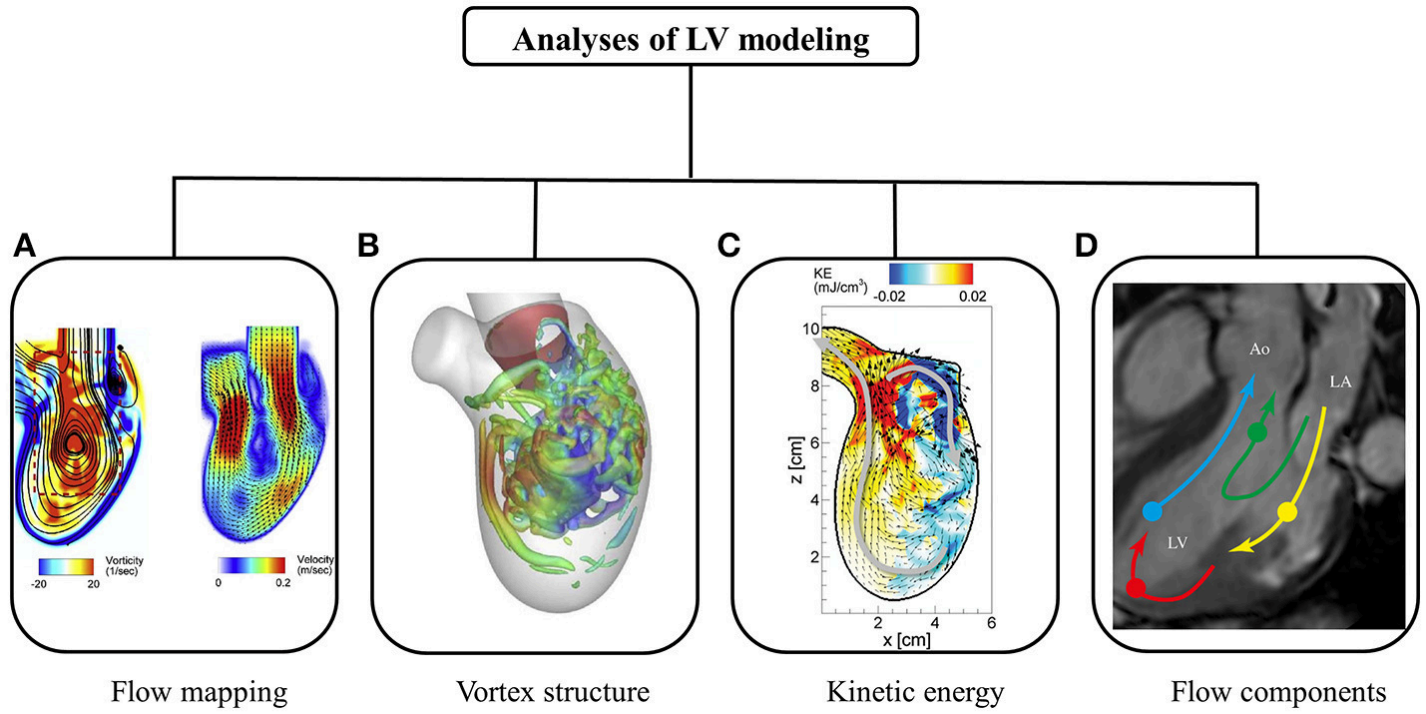
Post-processing of intra-cardiac flow showing **(A)** flow mapping (Seo et al., 2014); **(B)** vortex structure (Seo et al., 2014); **(C)** kinetic energy (Seo and Mittal, 2013); and **(D)** flow component (Svalbring et al., 2016).

Figure 5 summarizes various post-processing analyses of intra-cardiac flow. The flow mapping facilitates visualization of flow patterns throughout the cardiac cycle. Characteristic of diastolic flow is the clockwise anterior vortex, which is believed to preserve momentum and direct the flow toward the outflow tract (Chnafa et al., 2014). The flow mapping depicts one 2D slice of the complex 3D LV flow at a time. Therefore, 3D vortex structure is superior, and has been widely applied in the literature.

In a normal subject, a vortex ring is observed during the rapid filling phase, which results from flow separation at the mitral valve tip (Seo et al., 2014). The vortex ring interacts with endocardium and dissipates (i.e., breaking into smaller eddies during diastasis). Another vortex ring is again generated during atrial contraction, albeit weaker in terms of penetration distance. Both 2D flow mapping and 3D vortex structure provide quantitative analyses of LV flow.

A number of parameters have been proposed to quantify kinetic energy and energy loss during cardiac motion. Pumping efficiency during systole is defined as the ratio of total flux of mechanical energy through the aorta and power expended on blood flow by heart muscle (Seo et al., 2014). In contrast, myocardial efficiency based on the pressure-volume loop is defined as the ratio of systole work and energy consumption of heart muscle. Kinetic energy dissipation is closely related to flow field, and is the energy dissipated into heat. Relatively higher energy dissipation is expected in regions with complex flow fields due to vortex formation. Since energy dissipation is sensitive to flow field, it has been used to diagnose ventricular dysfunction (Mangual et al., 2013).

To quantify blood transit across the LV, numerous trajectories of massless particles are obtained over a few cardiac cycles (Pedrizzetti and Domenichini, 2014). According to flow trajectories, end-diastolic volume is categorized into volumes of four subgroups: (i) direct flow, blood that passes through ventricle during one heartbeat; (ii) retained flow, blood that enters during diastole but does not exit at end of systole in one heartbeat; (iii) delayed ejection flow, blood that enters ventricle in earlier cardiac cycles but exits in current cycle; and (iv) residual volume, blood that resides in ventricle over a number of cardiac cycles. *In-vivo* studies demonstrate direct and residual flow (≥2 cycles) constitute about 37 and 30%, respectively, of end-diastolic volume, respectively (Eriksson et al., 2011). Although it is believed that larger residual volume may promote thrombogenesis, clinical significance of the relative distribution of the four volumes of subgroups has not been elucidated. The considerable differences among numerical studies (Mangual et al., 2013; Seo and Mittal, 2013) could be due to uncertainties, differing assumptions and the methodologies adopted.

### Challenges and opportunities in use of patient-specific CFD technologies for diagnosing heart dysfunction

A promising application of numerical models is found in surgical and interventional planning for predicting procedural outcomes. Corsini and coworkers compared two surgical options for a patient with single ventricle malformation (Corsini et al., 2014). A secondary application is to advance the understanding of the effects of myocardial disease and surgery on ventricular flow. Su and colleagues deduced that HCM retarded the formation of vortex ring during diastole because the narrowed LV chamber delayed flow separation and escalated energy dissipation (Su et al., 2014a). Although various effects of myocardial disease on ventricular flow have been investigated, sample sizes in these numerical studies were too small to provide meaningful insights.

**Figure 6:**
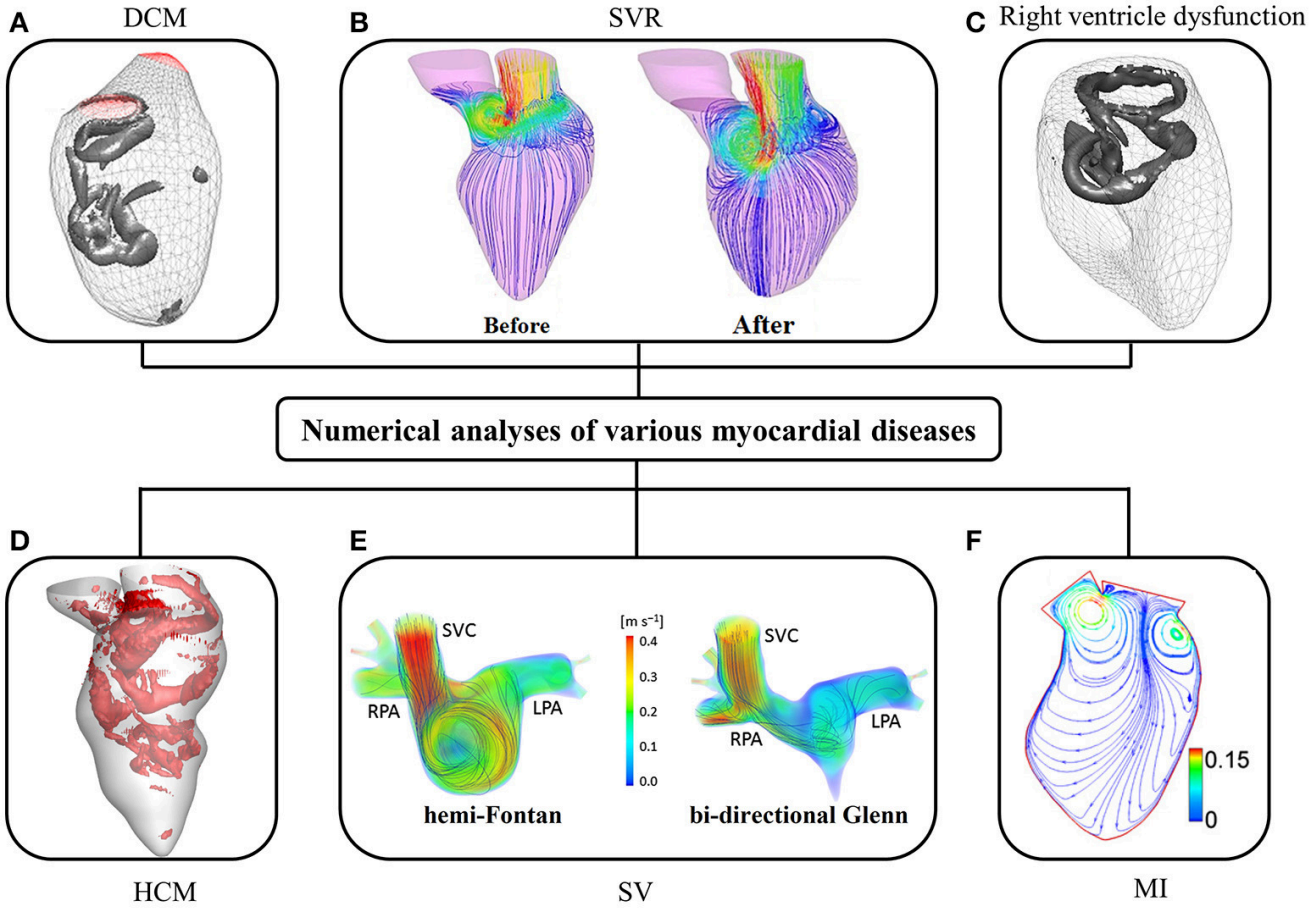
CFD simulation of intra-cardiac flow showing **(A)** vortex formation in dilated cardiomyopathy (DCM) (Mangual et al., 2013); **(B)** the distribution of blood velocity before and after surgical ventricular restoration (SVR) (Khalafvand et al., 2014); **(C)** vortex formation in right ventricle (Mangual et al., 2012); **(D)** vortex formation in hypertrophic cardiomyopathy (HCM); **(E)** distribution of blood velocity in a single ventricle (SV) (SVC, superior vena cava; RPA, right pulmonary artery; LPA, left pulmonary artery); **(F)** the distribution of blood velocity in the LV with myocardial infarction (MI) (Khalafvand et al., 2012).

Since the 1990s, numerical studies have been conducted to study heart function (Hunter et al., 2003; Sugiura et al., 2012) including intraventricular flows. The early studies were focused on ideal models due to limitations of imaging techniques, particularly for segmenting images from noisy data. More contemporary published patient-specific numerical studies are summarized in Table 4, in which the majority of studies focus on the LV rather than the RV (where the geometry is more complex than LV). As shown in Table 4, very few studies employed reconstructed 3D models from echocardiographic images (Mangual et al., 2013). Among imaging modalities, MRI is the most widely used owing to superior soft tissue contrast, and absence of ionizing radiation. In our experience, the accuracy of current commercial (e.g., 3D Doctor, Mimics, SliceOmatic, Amira) and open (e.g., ITK, ITK-SNAP, VTK, Analyze) segmentation software packages depends on the experience of the user who processes the MRI images. This is a hurdle to wider adoption of numerical studies. Most studies focus on normal subjects, with relatively few myocardial disease cases, for example, right ventricle (RV) dysfunction (Mangual et al., 2012), dilated cardiomyopathy (Mangual et al., 2013), hypertrophic cardiomyopathy (HCM) (Su et al., 2014a), single ventricle (Corsini et al., 2014), and myocardial infarction (Khalafvand et al., 2012) (Figure 6).

**Table 4:** Published patient-specific CFD simulations of heart ventricles.

**First author and year**	**Chamber**	**Imaging**	**Normal**	**PAH**	**DCM**	**SVR**	**SV**	**HCM**	**MI**	**MS**	**Valve**	**Dimensions**	**Method**
Doost et al., 2017	LV	MRI	1	–	–	–	–	–	–	–	N	2D	DM
Imanparast et al., 2016	LV	MRI	1	–	–	–	–	–	–	–	N	3D	DM
Su et al., 2016	LV	MRI	1	1	–	–	–	–	–	–	Y	3D	DM
Doost et al., 2016	LV	MRI	1	–	–	–	–	–	–	–	N	3D	DM
Chnafa et al., 2016	LV	CT	1	–	–	–	–	–	–	–	Y	3D	DM
Bavo et al., 2016	LV	Echo	2	–	1	–	–	–	–	–	Y	3D	DM
Vedula et al., 2015	LV	CT	1	–	–	–	–	–	–		Y	3D	DM
Su et al., 2014a	LV	MRI	1	–	–	–	–	1	–	–	N	3D	DM
Su et al., 2014b	LV	MRI	1	–	–	–	–	–	–	–	N	2D	DM
Khalafvand et al., 2014	LV	MRI	–	–	–	1	–	–	–	–	N	3D	DM
Moosavi et al., 2014	LV	MRI	1	–	–	–	–	–	–	–	N	3D	DM
Seo et al., 2014^#^	LV	CT	1	–	–	–	–	–	–	–	Y	3D	IBM
Chnafa et al., 2014^*^	LV	CT	–	–	–	–	–	–	–	–	Y	3D	DM
Corsini et al., 2014	RV	MRI	–	–	–	–	1	–	–	–	N	3D	DM
De Vecchi et al., 2014	LV/RV	Echo	–	–	–	–	1	–	–	1	N	3D	DM
Seo and Mittal, 2013^#^	LV	CT	1	–	–	–	–	–	–	–	N	3D	IBM
Mangual et al., 2013	LV	Echo	20	–	8	–	–	–	–	–	N	3D	IBM
Nguyen et al., 2013	LV	MRI	1	–	–	–	–	–	–	–	N	3D	DM
Le and Sotiropoulos, 2012	LV	MRI	1	–	–	–	–	–	–	–	N	3D	IBM
Mangual et al., 2012	RV	Echo	1	–	–	–	–	–	–	–	N	3D	IBM
Dahl and Vierendeels, 2012	LV	Echo	1	–	–	–	–	–	–	–	Y	2D	DM
Khalafvand et al., 2012	LV	MRI	3	–	–	–	–	–	3	–	N	2D	DM
Tay et al., 2011	LV	MRI	1	–	–	–	–	–	–	–	N	3D	IBM
Mihalef et al., 2011	LV/RV	CT	–	–	–	–	–	–	–	1	Y	3D	DM
Krittian et al., 2010	LV	MRI	1	–	–	–	–	–	–	–	N	3D	DM
Doenst et al., 2009	LV	MRI	1	–	–	1	–	–	–	–	N	3D	IBM
Schenkel et al., 2009	LV	MRI	1	–	–	–	–	–	–	–	N	3D	DM
Long et al., 2008	LV	MRI	6	–	–	–	–	–	–	–	N	3D	DM
Saber et al., 2003	LV	MRI	1	–	–	–	–	–	–	–	N	3D	DM
Saber et al., 2001	LV	MRI	1	–	–	–	–	–	–	–	N	3D	DM

*PAH, Pulmonary Arterial Hypertension; DCM, Dilated Cardiomyopathy; SVR, Surgical Ventricular Restoration; SV, Single Ventricle; HCM, Hypertrophic Cardiomyopathy; MI, Myocardial Infarction; MS, Mitral Stenosis; DM, Dynamic Mesh; IBM, Immersed Boundary Method*;

* *The type of heart disease was not specified in the manuscript*;

# *Only the initial shape is based on patient-specific data*.

Although valve leaflet dynamics influence intraventricular flow development relative to vortex formation mechanisms and penetration depth, they are not taken into consideration in most studies as current spatiotemporal resolution of MRI and CT images are inadequate for assessing the rapid opening and closing of thin heart valves. Although the mitral valve was incorporated by Seo et al. (2014), the model was not a full patient-specific model. Only the initial geometry was based on patient-specific CT data, while the remaining geometries were simply dilated according to an ideal model. In addition, the mitral valve motion was pre-defined rather than based on patient-specific data. Mihalef et al. (2011) studied a patient with severe mitral stenosis and regurgitation. The expected strong forward jet toward the apex during diastole and the reversal toward the atrium during systole balanced out, preempting a mismatch between the leaflet dynamics and LV volumetric changes in this selected case.

One feasible solution to model valvular motion is the FSI (Khalafvand et al., 2011; Domenichini and Pedrizzetti, 2014; Doost et al., 2016), which to date has been applied only in 2D studies (Table 4). Basically, there are two methods for modeling myocardium deformation during a cardiac cycle: dynamic mesh and immersed boundary method. Volumetric mesh deforms to cope with the motion of the boundary (e.g., the ventricle) in the dynamic mesh method; while in the immersed boundary method, modeling is accomplished using stationary grids and adding force near the boundary in the Navier-Stokes equations to take ventricular wall effects into account. Although the immersed boundary method avoids the issues of potential meshing failure during mesh generation and deformation, additional functions must be added to obtain the solution, which results in extra computational cost.

## Conclusions

Regulatory authorities such as US Food and Drug Administration have recognized the value of computer modeling and simulation in the regulatory approval process (Malinauskas et al., 2017). Authorities encourage use of the simulation to complement bench, animal and human testing. There are an increasing number of FDA applications that include simulations. The Heart Flow's FFR_CT_ software has been approved by the US FDA for measuring coronary blockages non-invasively. Because most healthcare practitioners and organizations are unfamiliar to the technical, computational and simulation methodologies of patient-specific CFD, and the methodology is not yet fully developed, there is an understandable hesitancy to embrace the approach. This is in addition to the presently unrealized ability of researchers and practitioners advocates to effectively communicate the potential benefits of patient-specific CFD. One possible reason for the lack of interest by clinicians could be the lack of a validation protocol in general. A general validation protocol would stipulate procedures and methods for measuring a specified clinical quantity using a standard technique and comparing it to a CFD computation. However, the virtual absence of specific clinical quantities with recognized links to the vast majority of pathologies—with the exception of FFR for PCI—upon which decisions could be predicted, creates a particularly challenging obstacle in the validation of patient-specific models. In addition, development of patient-specific models is a time-consuming task that requires patient-specific geometries, material properties, and realistic boundary conditions. These represent formidable challenges, but at the same time significant opportunities to interject precision medicine into clinical practice for improved clinical outcomes.

## Author contributions

LZ, J-MZ and BS: conception or design of the work; LZ, J-MZ, BS, RT, JA, and GK: draft the work or revise it critically for important intellectual content. All authors have seen and approved the final version of manuscript.

### Conflict of interest statement

The authors declare that the research was conducted in the absence of any commercial or financial relationships that could be construed as a potential conflict of interest.
